# Clinical-Pathological Conference Series from the Medical University of Graz: Case No 173: A 77-year-old patient with adenocarcinoma of the prostate, liver metastases and watery diarrhea

**DOI:** 10.1007/s00508-020-01791-x

**Published:** 2021-01-04

**Authors:** Elisabeth Fabian, Patrizia Kump, Dietmar Schiller, Iva Brcic, Christine Gruber, Philipp U. Heitz, Günter Klöppel, Rainer W. Lipp, Farid Moinfar, Rainer Schöfl, Peter Fickert, Guenter J. Krejs

**Affiliations:** 1 Division of Gastroenterology and Hepatology, Department of Internal Medicine III, Medical University of Vienna, Vienna, Austria; 2 Division of Gastroenterology and Hepatology, Department of Internal Medicine, Medical University of Graz, Auenbruggerplatz 15, 8036 Graz, Austria; 3 Department of Internal Medicine IV, Ordensklinikum Barmherzige Schwestern, Linz, Austria; 4 Institute of Pathology, Medical University of Graz, Graz, Austria; 5 Institute of Clinical Pathology, Vinzenz Pathologie Verbund, Linz, Austria; 6 Department of Pathology, University of Zurich, Zurich, Switzerland; 7 Institute for General Pathology, Technical University of Munich, Munich, Germany; 8 Division of Oncology, Department of Internal Medicine, Medical University of Graz, Graz, Austria

**Keywords:** VIPoma, Pancreatic cholera, Verner-Morrison syndrome, WDHH (watery diarrhea, hypokalemia, hypochlorhydria) syndrome, Hypokalemia

## Presentation of case

### Dr. D. Schiller:

The patient was admitted because of watery diarrhea persisting for 2 months. He had been diagnosed with a grade 3 (G3) adenocarcinoma of the prostate 18 months earlier, Gleason score 9 (4 + 5) with blockage of the ureters leading to bilateral hydronephrosis and kidney failure. In addition to prostate cancer, computed tomography (CT) revealed extensive retroperitoneal lymphadenopathy and disseminated bone metastases, whereas the liver, spleen, pancreas, mediastinum and both lungs were unremarkable. Serum prostate-specific antigen (PSA) was over 1000 ng/mL (normal: <6.5 ng/mL). After transurethral resection of the prostate (TURP), bilateral ureteral splinting and nephrostomy of the right kidney, serum creatinine decreased from 2.4 to 1.3 mg/dL (normal: 0.7–1.2 mg/dL). Serum electrolytes were all within normal limits. The oncological management included an initial dose of bicalutamide and continuous therapy with leuprolide acetate administered intramuscularly every 3 months. In addition, the patient was given denosumab subcutaneously once a month. While on this therapy, the PSA levels returned to normal and the enlarged lymph nodes markedly decreased in size. Subsequently, the bilateral ureteral splints could be removed and the patient became free of symptoms; however, about 2 months before the current admission he started to have watery diarrhea, which also persisted during the night. He did not complain of abdominal pain or reduced appetite. Ileocolonoscopy with multiple biopsies and stool cultures yielded unremarkable results. Except for fluid-filled small bowel loops, CT of the abdomen and chest did not reveal new findings. Watery diarrhea was associated with hypokalemia, and the administration of loperamide and opium tincture had no effect on the diarrhea. A therapeutic trial with ciprofloxacin and metronidazole was also ineffective.

On admission, the patient with a body weight of 76 kg and body height of 185 cm appeared dehydrated. He was afebrile and his blood pressure was 110/70 mm Hg. The nephrostomy scar on his right flank was unremarkable, fecal occult blood test was negative and the physical examination was otherwise unrevealing. Apart from adenocarcinoma of the prostate, the patient’s history was negative for chronic diseases. He was not on any long-term medication, and his travel history and family history were negative.

Laboratory data: Leukocytes 12.8 G/L (normal: 4.4–11.3 G/L) with unremarkable differential count, hemoglobin 10.0 g/dL (normal: 12.0–15.3 g/dL), platelets 685 G/L (normal: 140–440 G/L). Urinalysis showed 75 mg protein/dL and sporadic erythrocytes. Serum sodium 131 mmol/L (normal: 135–145 mmol/L), potassium 2.1 mmol/L (normal: 3.6–4.8 mmol/L), blood urea nitrogen (BUN) 64 mg/dL (normal: 8–23 mg/dL), creatinine 3.8 mg/dL (normal: 0.7–1.2 mg/dL), total protein 7.0 g/dL (normal: 6.4–8.3 g/dL), albumin 3.1 g/dL (normal: 3.5–5.2 g/dL), lactate dehydrogenase (LDH) 522 U/L (normal: <248 U/L), alkaline phosphatase 263 U/L (normal: 30–120 U/L), gamma-glutamyl transferase (GGT) 58 U/L (normal: <60 U/L), C‑reactive protein (CRP) 3.0 mg/dL (normal: <0.5 mg/dL), chromogranin A 22 nmol/L (normal: <10 nmol/L), free triiodothyonine (fT3) 2.8 pmol/L (normal: 3–7.6 pmol/L), free thyroxine (fT4) 13 pmol/L (normal: 10–28 pmol/L), thyroid-stimulating hormon (TSH) 0.01 mU/L (normal: 0.35–4 mU/L). Serum aspartate aminotransferase (AST) and serum alanine aminotransferase (ALT), calcium, phosphate, glucose, immunoglobulins (Ig) G, IgA, IgM, amylase, tissue transglutaminase antibodies, gastrin, calcitonin, PSA, calprotectin in stool and 5‑hydroxy-indole acetic acid (5-HIAA) in a 24‑h urine collection were all normal. Urinary potassium excretion was 13 mmol/24 h. Immunofixation in serum, light-chain analysis in urine, human immunodeficiency virus (HIV) serology, analysis of HLA-DQ2 and DQ8, as well as microscopic screening of three stool samples for ova and parasites were all normal or negative.

Stool volume was not measured but was documented as high output even during fasting. Under intravenous rehydration therapy with up to 4 L per day and substitution of 140 mmol potassium per day, serum potassium only reached 2.8 mmol/L and serum creatinine decreased to 1.6 mg/dL.

Esophagogastroduodenoscopy (EGD) with biopsies including the second portion of the duodenum revealed reflux esophagitis grade II according to Savary-Miller. Intravenous administration of a proton pump inhibitor as well as therapy with doxycycline and octreotide failed to relieve the patient’s diarrhea. Congo red staining and Whipple PCR of the colonic and duodenal mucosa were negative. Magnetic resonance (MR) enteroclysis showed fluid-filled small bowel loops and the already known retroperitoneal lymphadenopathy, but no masses in the liver, pancreas or spleen.

During the further course, the patient developed atrial fibrillation with a rapid ventricular response and sepsis from a central venous catheter. He was transferred to the intensive care unit but then refused further work-up and therapy. The patient died after 5 weeks of best supportive care.

Autopsy revealed enlarged retroperitoneal lymph nodes, disseminated bone metastases and remnant prostatic tissue. Except for two 8 mm white lesions in the liver, all other visceral organs were unremarkable.

A diagnostic result became available.

## Differential diagnosis

### Dr. P. Kump:

The patient under discussion is a 77-year-old man with adenocarcinoma of the prostate (G3), retroperitoneal lymphadenopathy and bone metastases, who developed postrenal kidney failure treated with TURP, bilateral ureteral splinting and nephrostomy of the right kidney. The oncological management included bicalutamide and leuprolide acetate; furthermore, he was given denosumab. This therapy rendered the patient stable and free of symptoms. He started to complain of watery diarrhea 2 months before the current admission, which also persisted during the night. On admission, the patient presented with dehydration and hypokalemia. Renal function parameters, i.e. creatinine and BUN, were increased and mild hypoalbuminemia was observed. Although the patient was dehydrated, his blood count showed anemia. Markedly increased levels of alkaline phosphatase and LDH were most likely due to the patient’s metastatic prostate cancer. Urinalysis showed reduced excretion of potassium and proteinuria. Calculation of serum osmolality (372 mosmol/kg, normal: 275–305 mosmol/kg) revealed hyperosmolality, which is frequently found in renal failure and diarrhea, as present in the discussed patient. In summary, laboratory data reflect diarrhea with dehydration and postrenal kidney failure, gastrointestinal loss of potassium, proteinuria due to dehydration, anemia most likely associated with cancer, and evidence of bone metastases.

One of the key symptoms in the discussed patient is a 2-month history of watery diarrhea (4–6 weeks of diarrhea is defined as chronic diarrhea), which may be due to osmotic, secretory, functional or inflammatory causes; however, since endoscopy with biopsies was unremarkable in the discussed patient diagnoses, such as celiac disease, microscopic colitis, inflammatory bowel disease and eosinophilic enteritis can be ruled out. Normal levels of tissue transglutaminase antibodies and the analysis of HLA-DQ2 and DQ8 confirmed the absence of celiac disease as did normal calprotectin in stool for inflammatory bowel disease. Biopsies negative for the Congo red stain excluded amyloidosis. Normal serum levels of calcium, fT4 and calcitonin make the diagnosis of hyperparathyroidism and medullary thyroid carcinoma unlikely. Acquired immune deficiency syndrome (HIV enteropathy), carcinoid syndrome and gastrinoma can also be excluded due to negative HIV serology, normal levels of 5‑HIAA in 24‑h urine and normal serum gastrin, respectively. Diarrhea caused by a surgically altered gastrointestinal tract, radiation or laxative abuse can also be ruled out in this case because of the negative history. This suggests that functional and inflammatory reasons for chronic diarrhea are unlikely in this patient; however, osmotic and secretory reasons are candidates and should be discussed in detail. Osmotic diarrhea occurs when too many solutes such as lactose, magnesium salts or some artificial sweeteners stay in the intestine and water cannot be absorbed properly, consequently causing a high fecal output. Thus, osmotic diarrhea always depends on what has been ingested previously and therefore, typically subsides during nighttime and on fasting. Secretory diarrhea occurs when the intestine actively secretes electrolytes followed by water. Diarrhea during the night might reflect an imbalance between secretion and reabsorption processes. Nocturnal diarrhea in the discussed patient thus strongly suggests secretory diarrhea, most likely as an adverse drug reaction, or caused by a neuroendocrine tumor producing a secretagogue, such as vasoactive intestinal polypeptide (VIP). The patient was receiving therapy with bicalutamide, leuprolide acetate and denosumab, which are all drugs commonly associated with gastrointestinal side effects including diarrhea according to the consumer medical information [1–3]. Since the patient had been on these medications for a long time without side effects, however, it seems unlikely that his current condition is drug-associated, but it cannot be completely excluded as a differential diagnosis.

VIPoma syndrome, also termed pancreatic cholera or Verner-Morrison syndrome, is due to a neuroendocrine tumor that secretes VIP, leading to watery diarrhea, hypokalemia, hypochlorhydria and metabolic acidosis. VIP is a neurotransmitter in the central and peripheral nervous system, and particularly in the peptidergic nervous system [4]. A low plasma level of VIP is normal and considered a neuronal overflow. Elevated serum levels induce intestinal secretion comparable to cholera toxin, thus leading to extensive watery diarrhea [5]. In adults, VIPomas are typically located in the pancreas as solitary tumors, with a mean size range of 4.5–5.4 cm [6]. Extrapancreatic VIPomas are rare and usually occur in children. They are neurogenic tumors arising from the sympathetic paraganglia and adrenal glands. Rarely, VIPomas have also been reported in patients with watery diarrhea, hypokalemia, hypochlorhydria (WDHH) syndrome and multiple small and large pancreatic neuroendocrine tumors as part of the multiple endocrine neoplasia type I (MEN I) syndrome [7]. In the patient under discussion, CT revealed extensive retroperitoneal lymph node metastases and disseminated bone metastases, but failed to identify any tumor in the liver, spleen, pancreas, mediastinum and lungs that could be considered a primary tumor. There was also no clinical or familial indication of a MEN1 syndrome. Autopsy confirmed the metastases seen on imaging, and in addition found two small tumors in the liver. If these are of neuroendocrine nature, a pancreatic or extrapancreatic VIPoma metastasizing to the liver and giving rise to the WDHH syndrome appears to be unlikely. Instead, it is conceivable that cells in the poorly differentiated prostate carcinoma had developed an independent neuroendocrine clone that spread to the liver.

The majority of prostate cancers have the phenotype of adenocarcinoma and require hormonal exposure to gonadal androgen for cell growth. Inhibition of the androgen receptor signaling pathway by androgen deprivation therapy is therefore the standard of care. In most cases, however, the primary androgen deprivation therapy is only transiently effective and prostate cancer progresses after a variable period of time to a status known as castration-resistant prostate cancer (CRPC) [8], for which several different therapeutic options are available, such as chemotherapy with cabazitaxel [9], inhibition of androgen receptor by abiraterone [10] and enzalutamide [11], sipuleucel‑T immunotherapy [12], radiotherapy with radium-223 chloride [13] and taxane-based chemotherapy [14]. The transformation of CRPC into a neuroendocrine phenotype with low or zero androgen receptor expression in patients on androgen receptor-directed therapies has been increasingly recognized in clinical practice [15–17]. Furthermore, therapy-induced neuroendocrine differentiation has been reported for agents such as docetaxel [18, 19], enzalutamide and abiraterone [20, 21]. Moreover, radiotherapy has also been found to lead to neuroendocrine differentiation [22]; however, the mechanism of transformation is still incompletely understood. Normal prostate tissue consists of three types of epithelial cells: basal cells, luminal cells and neuroendocrine cells (<1%) [23]. In adenocarcinoma of the prostate, an increased number of neuroendocrine-like cells can be found [23–25] and it has been hypothesized that these cells arise from luminal-type prostate cancer cells by a neuroendocrine differentiation or transdifferentiation process. Neuroendocrine-like cells do not proliferate and lack expression of androgen receptor and PSA, making them resistant to therapy [26]. They also exhibit alterations in intracellular calcium homeostasis [27] and secrete a number of peptide hormones and growth factors to support the growth of surrounding tumor cells in a paracrine manner [26]. Neuroendocrine-like cells in adenocarcinoma and normal endocrine cells share many characteristics, but they also differ in various aspects. For example, normal neuroendocrine cells express basal cell markers, whereas neuroendocrine-like cells express luminal cell markers. It is suggested that stimulation of different complex signaling pathways through various therapeutic agents induces formation of neuroendocrine-like cells by a transdifferentiation process of prostate cancer cells, either from hormone-naive or CRPC cells [28]. Data emphasize that therapy-induced neuroendocrine differentiation is correlated with poor survival in CRPC patients [20, 21].

## Dr. P. Kump’s diagnosis

Verner-Morrison syndrome due to VIP production by liver metastases of neuroendocrine-differentiated prostate carcinoma after antiandrogenic therapy.

## Discussion of case

### Dr. I. Brcic:

**Fig. 1:**
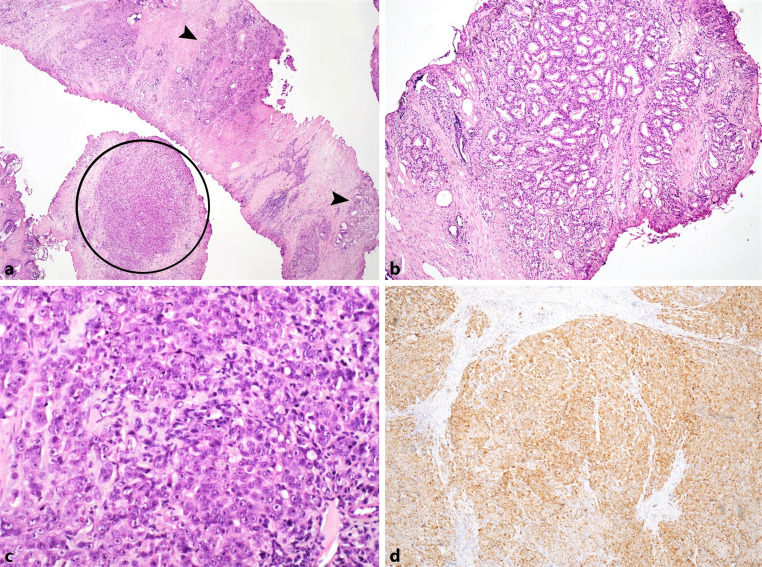
Morphological and immunohistochemical findings of the prostate tumor. **a** Two components are present: typical adenocarcinoma (*arrowheads*) and a poorly differentiated carcinoma (*circle*) (hematoxylin and eosin [H&E] ×20). **b** Typical adenocarcinoma is composed of irregular glandular structures (H&E ×40). **c** A poorly differentiated component composed of sheets of enlarged atypical cells with visible nucleoli (H&E ×400). **d** Immunohistochemical PSA staining shows diffuse expression (×400)

Histological examination of the removed prostate showed two components: (1) prostate adenocarcinoma composed of atypical glandular structures and (2) a poorly differentiated carcinoma comprised of enlarged atypical cells with visible nucleoli arranged in larger nests (Fig. 1a–c). Immunohistochemically, both components showed diffuse expression of PSA (Fig. 1d). These findings support the diagnosis of a poorly differentiated adenocarcinoma of the prostate with a Gleason score 9 (4 + 5).

**Fig. 2:**
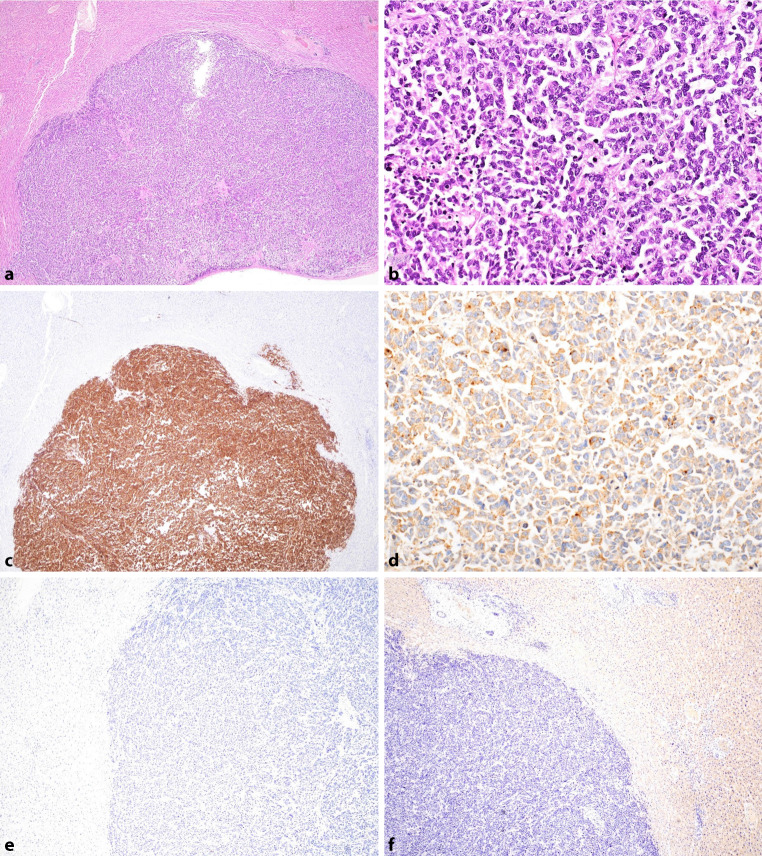
Morphological and immunohistochemical findings of the liver metastases. **a** Liver fragment with nodular tumor tissue (HE ×20). **b** Tumor is composed of diffuse sheets of tumor cells with irregular nuclei without nucleoli (HE ×400). **c** Immunohistochemistry shows positive expression of synaptophysin (×20) and **d** VIP (×200), and negativity for PSA (×40) (**e**) and prostein (×40) (**f**)

The liver metastases were composed of sheets of tumor cells with an endocrine phenotype (Fig. 2a, b) and mitotic activity. Immunohistochemistry showed labelling for synaptophysin and VIP (Fig. 2c, d). PSA, prostein and chromogranin A stains were negative (Fig. 2e, f). These findings suggested the diagnosis of well-differentiated neuroendocrine tumor metastases with VIP expression.

### Dr. G.J. Krejs:

Serum for VIP radioimmunoassay was not available in this patient so all emphasis lies on the liver lesions. With the findings Dr. Brcic described, we contacted two leading European experts in pathology of neuroendocrine tumors, Dr. Philipp U. Heitz of Zurich and Dr. Günter Klöppel of Munich.

### Dr. P.U. Heitz (by email):

The excellent histological sections provided by Dr. Brcic and the Linz pathologists show two tumors: an adenocarcinoma of the prostate, in part poorly differentiated and a neuroendocrine tumor in the liver. The liver tumor cells are strongly positive for synaptophysin, but negative for chromogranin. The difficult to obtain VIP phenotyping shows sufficient positivity to assume VIP production and release. I have seen numerous neuroendocrine phenotypic tumors in the prostate, but never a VIPoma which makes this an extremely unusual and rare case. The tumor in the prostate shows a morphological (in part adenocarcinoma, in part solid carcinoma) and immunophenotypic (PSA positive) pattern different from the tumor in the liver. This does not rule out that the tumor in the liver originates from the prostate, but it certainly is not very likely. Rather, the patient may have two separate malignant tumors, with the tumor tissue in the liver being morphologically and immunophenotypically a VIPoma.

### Dr. G. Klöppel (by email):

The prostate showed typical adenocarcinoma with a poorly differentiated part and large areas of solid tissue, Gleason scores 9 (4 + 5). Tissue sections were sent to us for further immunohistochemical examination. The tumor of the prostate is diffusely positive for NKX3.1 (a prostate marker) and displays single-cell nuclear positivity for synaptophysin, but negativity for VIP and Islet‑1, a marker for pancreatic neuroendocrine tumors (PanNET). The liver lesions are metastases of a neuroendocrine tumor, positive for VIP and synaptophysin, but negative for NKX3.1. The tumor tissue in the liver does not originate from a PanNET since the immunohistochemistry for Islet‑1 is negative. Thus, the origin of the liver metastases remains unclear. One can speculate that they arose from the poorly differentiated solid part of the prostate cancer and lost prostate-specific identity in the process of metastasizing. Chemotherapy may have played a role in this. The pancreas, which is the usual origin of VIPomas, can be ruled out as the source as described by the immunohistochemical findings; moreover, the autopsy did not show any tumor in the pancreas. The neuroendocrine tumor tissue in the liver with VIP expression explains the clinical presentation as Verner-Morrison syndrome and classifies the tumor as a VIPoma.

### Dr. G.J. Krejs:

Both experts independently classified the liver tumor as VIPoma. In the history of the clinical-pathological conferences at the Medical University of Graz, out of 172 previous cases, 1 patient with a VIPoma had been discussed by Dr. John Fordtran from Dallas, Texas in 1989. VIP is a 28 amino acid polypeptide with a molecular weight of 3326 [29] which was initially characterized by Said and Mutt in 1968 [30, 31] as a vasoactive substance and later suggested to be a candidate gastrointestinal hormone [32]. In 1976, when the peptide was demonstrated in central and peripheral nervous systems, it became apparent that the major function of VIP may be that of a neuropeptide, neurotransmitter or neuromodulator [4]. The association between pancreatic tumor and watery diarrhea was first described by Verner and Morrison [33], who could distinguish their cases with low gastric secretion from gastrinoma (Zollinger-Ellison syndrome) described 3 years earlier [34]. In searching for a humoral mediator, Bloom et al. [35] in 1973 found VIP-containing cells in the tumor tissue and, using the newly available radioimmunoassay, high VIP plasma concentrations in affected patients. This finding was confirmed in 13 patients with pancreatic islet-cell tumors by Said et al. [36]. Gut endocrine tumors have an annual incidence of 3 per 100,000 population [37]; VIPomas can be expected at a yearly rate of 1 per 10 million population, i.e. not even 1 case per year in Austria [4]. The key clinical feature of VIPoma syndrome is large-volume secretory diarrhea with up to 9000 g stool per day (mean 4224 g) and persisting diarrhea with up to 3350 g per day (mean 1817 g) during fasting. These numbers are based on observations of a total of 12 patients. In secretory diarrhea stool water is isotonic to plasma and stool electrolytes account for all the osmolality [38]. Loss of large amounts of potassium and bicarbonate in stool results in hypokalemia, acidosis and volume depletion [4]. We measured an average of 70 mEq potassium per liter in stool water of those patients. Thus, total body potassium (about 3500 mEq) gets rapidly depleted. Achlorhydria or hypochlorhydria is also a characteristic feature of VIPoma syndrome but is not present in all patients [39]. Additional clinical features are listed in Table 1. A prolonged (10h) VIP infusion (400 pmol/kg/h) has been shown to mimic the syndrome in healthy subjects who all developed watery diarrhea and metabolic acidosis [40–42]. In a canine model, VIP administered via the mesenteric artery caused intestinal secretion in loops of jejunum similar to cholera toxin [43]. Administration of somatostatin can decrease intestinal secretion by direct reduction of release of the secretagogue from the tumor [42, 44]. The average duration of symptoms in VIPoma syndrome is 3 years prior to diagnosis, with a range from 2 months to 4 years. Metastases (liver, lymph nodes) are found in 50% of patients at the time of diagnosis. Death occurs from renal failure or cardiac arrest due to water and salt depletion, hypokalemia and acidosis [4].

**Table 1:** Clinical features of VIPoma syndrome [4]

*Constant features*	Diarrhea
	Hypovolemia
	Acidosis
	Hypokalemia
*Variable features*	Achlorhydria or hypochlorhydria
	Hypercalcemia
	Hypomagnesemia
	Enlarged gallbladder
	Myopathy or nephropathy (hypokalemic)
	Rash
	Flushing
	Hyperglycemia
	Lacrimal gland hypersecretion and excessive tearing

A VIPoma is typically associated with an islet-cell tumor; in the discussed patient, however, no pancreatic mass was found on autopsy. This suggests that, as outlined by Dr. Kump, neuroendocrine tumor tissue has differentiated from the patient’s prostate cancer.

Although VIPoma is a rare disease, it should be considered as a cause of large-volume diarrhea among other diagnoses, such as laxative abuse, malignant carcinoid syndrome, calcitonin excess and celiac disease, particularly when hypokalemia is present. When large-volume diarrhea and a pancreatic mass are encountered, practically only two differential diagnoses, i.e. VIPoma and gastrinoma are left. Although both diseases may present with severe diarrhea, the underlying pathophysiology is completely different due to the effects mediated by gastrin and VIP. Voluminous gastric secretion due to gastrin overwhelms the absorptive capacity of the intestine while VIP causes intestinal net fluid gain due to active mucosal secretion [45].

### Dr. G.J. Krejs:

Let me ask Dr. Lipp, chairman of our Graz neuroendocrine tumor board, what today’s therapy would be if the diagnosis had been made while the patient was still alive.

### Dr. R.W. Lipp:

In the case that the patient would have gained initial symptom control, the recommended next treatment step should be the application of a long-acting somatostatin analogue, such as octreotide or lanreotide, which blocks the release of VIP from tumor tissue and improves symptoms [44]. It has also shown some antiproliferative and antitumor effects in patients with gastroenteropancreatic NETs [46, 47]. Nearly all patients with VIPoma show high somatostatin receptor expression in tumor cells. This allows easy visualization with ^68^Ga-DOTA-NOC positron emission tomography (PET)/CT with higher sensitivity and specificity as compared to CT or MR imaging [48, 49]. If the tumor has not spread to other organs, surgery would be the first choice. For metastatic disease in the presence of a nearly normal kidney function and satisfactory red and white blood cell counts, peptide radioligand therapy with a beta-emitting radionuclide-labeled somatostatin analogue, such as ^177^Lu-DOTA-TATE would be a second-line therapeutic option. The prospective, phase III NETTER‑1 trial has shown in patients with gastroenteric midgut NETs and progressive disease at study entry, a risk reduction of tumor progression in up to 79% as compared to octreotide-LAR 60 mg [50]. In patients with VIPoma refractory to somatostatin analogues, other treatment options (however, with only limited evidence from prospective studies) would include glucocorticoids, interferon-alpha, everolimus and sunitinib [51, 52]. Systemic chemotherapy such as the doxorubicin/streptozocin or temozolomide-based regimens should be used only in patients with progressive bulky tumors or patients with widespread disease [53].

### Dr. P. Kump:

This is a very interesting and instructive case of a patient with VIPoma syndrome due to a neuroendocrine tumor, most likely a neuroendocrine-differentiated prostate cancer caused by antiandrogenic therapy. In my experience an estimated 20% of patients with NETs have a second tumor in their history with prostate cancer being one of the most frequently observed entities.

## Final diagnosis

VIPoma, also termed pancreatic cholera, Verner-Morrison syndrome or WDHH (watery diarrhea, hypokalemia, hypochlorhydria) syndrome due to VIP-expressing tumor tissue in the liver with unknown primary but most likely originating from neuroendocrine differentiation in posttreatment prostate cancer.
